# Effect of l‐Cysteine on Enzymatic Browning, Antioxidant Activity, and GSH Stability in Fresh‐Cut Quince: A Comparative Evaluation With Potassium Metabisulfite

**DOI:** 10.1002/fsn3.72113

**Published:** 2026-07-13

**Authors:** Omca Demirkol, İnci Cerit, Özlem Aktürk Gümüşay

**Affiliations:** ^1^ Department of Food Engineering Sakarya University Sakarya Turkey; ^2^ Department of Gastronomy and Culinary Arts Maltepe University İstanbul Turkey

**Keywords:** antioxidant activity, enzymatic browning, fresh‐cut quince, glutathione, l‐cysteine, polyphenol oxidase (PPO)

## Abstract

This study evaluates the effectiveness of l‐cysteine and sodium metabisulfite (1% solutions) in reducing enzymatic browning and preserving antioxidant properties in fresh‐cut quince slices during 9 days of cold storage (4°C). Key quality parameters assessed included color values (L*, a*, b*), polyphenol oxidase (PPO) activity, total phenolic content (TPC), glutathione (GSH) levels, and antioxidant capacity (via DPPH and FRAP assays). Results show that l‐cysteine was more effective than sodium metabisulfite in maintaining color brightness (higher L* values), suppressing red‐brown discoloration, and enhancing antioxidant activity in the early storage period. l‐cysteine acts by inhibiting PPO and scavenging free radicals, thereby reducing the formation of brown pigments and preserving phenolic compounds. However, in terms of GSH preservation, sodium metabisulfite performed better. The rapid loss of GSH in both l‐cysteine‐treated and untreated samples indicates that it undergoes rapid conversion to maintain cellular redox balance or that the 1% application of l‐cysteine—which possesses pro‐oxidant properties—is not solely used for the preservation of GSH. Prioritizing the elimination of redox imbalance in the environment suggests that there is a limitation of l‐cysteine's effect over time. Overall, l‐cysteine presents a promising alternative to sulfite‐based preservatives in fresh‐cut fruit preservation, demonstrating strong browning inhibition and antioxidant protection—particularly, for short‐ to mid‐term storage—while offering a potentially lower risk of health concerns associated with sulfites.

## Graphical abstract

This study investigates the effectiveness of l‐cysteine and sodium metabisulfite (1% solutions) in reducing enzymatic browning and preserving antioxidant properties in fresh‐cut quince slices stored at 4°C for 9 days. l‐cysteine proved more effective than sodium metabisulfite in maintaining color brightness and enhancing antioxidant activity by inhibiting polyphenol oxidase (PPO) and scavenging free radicals. However, sodium metabisulfite better preserved glutathione (GSH), indicating a complex interaction with cellular redox balance. Overall, l‐cysteine shows promise as a safer alternative to sulfite preservatives for the short‐ to mid‐term preservation of fresh‐cut fruits.

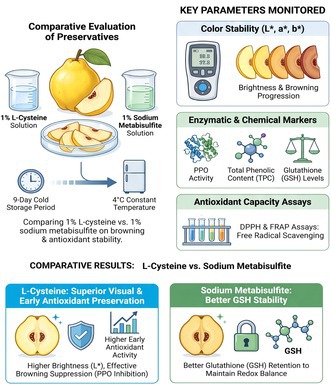

## 1 Introduction

Fresh‐cut fruits are increasingly popular in the market due to their convenience, but they are highly susceptible to quality degradation during postharvest storage. Enzymatic browning is one of the most critical phenomena contributing to this degradation, leading to undesirable discoloration, diminished nutritional value, and reduced consumer acceptance. Enzymatic browning primarily occurs through the oxidation of phenolic compounds, a process catalyzed by enzymes such as polyphenol oxidase (PPO). Upon mechanical damage, such as cutting, the activated PPO oxidizes phenolic compounds into quinones, which subsequently polymerize to form brown pigments (melanins) (Robles‐Sánchez et al. 2009; Zhu et al. 2022). Such effects have been reported in various fresh‐cut fruits, including bananas and apples, where the enzymatic activities of PPO and peroxidase (POD) contribute to the discoloration process (Mirshekari et al. 2017; Chen et al. 2021).

The quality degradation of fresh‐cut fruits encompasses not only enzymatic browning but also other factors such as texture loss and flavor changes. Mechanical processing can compromise the structural integrity of the fruit, leading to tissue softening and an increased surface area exposed to oxidative reactions (Liang et al. 2017). Furthermore, oxidative stress can deplete antioxidant compounds, further exposing fresh‐cut fruits to quality deterioration (Hu et al. 2022).

The sensory attributes of fresh‐cut fruits—specifically, their color, texture, and taste—are crucial determinants of consumer acceptance. Enzymatic browning directly impacts these sensory qualities, resulting in reduced market value. Studies emphasize that maintaining the esthetic and nutritional properties of fresh‐cut fruits is essential for sustaining consumer interest and sales (Cantos‐Villar et al. 2002; Wang et al. 2023).

Traditionally, sulfite‐based preservatives, such as sodium metabisulfite, have been employed to mitigate these effects through the inhibition of PPO and the reduction of oxidative stress (Seo et al. 2003; Fu et al. 2007). However, rising concerns regarding the allergenic and toxicological effects of sulfites have stimulated regulatory scrutiny and consumer demand for safer alternatives (Yang et al. 2001; Martine et al. 2025). In this context, l‐cysteine, a naturally occurring thiol‐containing amino acid, serves as an effective substitute for inhibiting PPO activity and reducing enzymatic browning in various fruits by preventing quinone formation (Yang et al. 2001; Lim et al. 2019). Specifically, studies have demonstrated that at concentrations of 1 mM, l‐cysteine effectively inhibits the activity of PPO in fruits like banana (Yang et al. 2001). Additionally, its interaction with quinones generated during enzymatic browning contributes to its effectiveness, as l‐cysteine can form stable, colorless products, preventing the undesirable brown pigments from developing (Sánchez‐Ferrer et al. 1993; Ali et al. 2014). Beyond its inhibitory effects on PPO, l‐cysteine plays a crucial role in the regeneration of endogenous antioxidants, such as glutathione (GSH). GSH is vital in cellular redox reactions and helps mitigate oxidative stress during storage (Martine et al. 2025). While sodium metabisulfite has been widely used for its strong inhibition of browning, studies indicate that l‐cysteine is potentially safer as a naturally occurring compound and offers comparable inhibition levels (Daroit et al. 2010; Ali et al. 2014). The effectiveness of l‐cysteine as an inhibitor has been confirmed in various studies; for instance, it inhibited PPO activity significantly more than some conventional methods (Cerit et al. 2020; Lim et al. 2019). Furthermore, given that sulfites can pose health risks for sensitive individuals, the adoption of l‐cysteine represents a shift toward potentially safer and more consumer‐friendly preservation methods (Yang et al. 2001; Molnár‐Perl and Friedman 1990; Martine et al. 2025).

Quince, a nutritionally important fruit valued for its distinctive aroma, high dietary fiber content, and richness in bioactive compounds, is also susceptible to enzymatic browning due to its high phenolic content and active oxidative enzymes (Wojdyło et al. 2013). Studies have been conducted on edible coating solutions to prevent the enzymatic browning of quince, and interpretations have been made based on color characteristics (Noshad et al. 2015; Salehi et al. 2023). Alternatively, studies have focused on products such as quince juice rather than directly on quince slices (Iqbal et al. 2018). This indicates that there are limited studies in the literature on fresh‐cut quince slices. Furthermore, no studies have been found specifically examining comparative antioxidant dynamics and GSH behavior.

In this study, quince ( *Cydonia oblonga* ) slices were treated with 1% l‐cysteine and 1% sodium metabisulfite solutions, followed by 9‐day storage at 4°C. At intervals of 0, 3, 6, and 9 days, key quality and antioxidant parameters were analyzed, including color attributes (L*, a*, b*), total phenolic content (TPC), PPO inhibition, GSH concentration, and antioxidant activities via DPPH and Ferric reducing antioxidant power (FRAP) assays. The primary objective was to evaluate the effectiveness of l‐cysteine compared to sulfite in suppressing enzymatic browning and maintaining antioxidant integrity in fresh‐cut quince. The intended preservation effect is specifically attributed to the fact that GSH levels, TPC, and antioxidant activity declines are minimized while L values, which measure brightness, remain high during the storage period.

This research contributes to the growing body of literature exploring natural and potentially safe alternatives to synthetic preservatives, addressing both functional efficacy and consumer safety in the postharvest handling of fresh‐cut produce.

## 2 Materials and Methods

### 2.1 Preparation of Fresh‐Cut Quinces and Antibrowning Solutions

Preliminary trials were conducted to determine the appropriate l‐cysteine concentration for fresh‐cut quince slices. Quinces obtained from local markets in Sakarya, Türkiye, were washed, peeled, and sliced into uniform thickness in ~2 cm discs. Each slice had an average weight of approximately 80 g. For each treatment and sampling period, analyses were performed using three slices as replicates. The slices were immersed for 1 min at room temperature in l‐cysteine solutions at concentrations of 0.05%, 0.1%, 0.5%, 0.8%, and 1.0%, while untreated samples served as controls. After draining for 30 min, samples were individually packaged in 50‐μm polyethylene bags and stored under refrigerated conditions (4°C). Color changes were evaluated after 9 days by calculating the total color difference (Δ*E*), as described in the color analysis section below. The Δ*E* values decreased with increasing l‐cysteine concentration, indicating improved inhibition of enzymatic browning (Table 1). The highest Δ*E* value was observed in the 0.05% treatment (23.89), whereas the lowest values were obtained for the 0.8% and 1.0% treatments (12.34 and 11.18, respectively). Concentrations above 1.0% were not evaluated because preliminary trials with higher concentrations resulted in an intense sulfite odor that was considered undesirable for sensory quality. Based on these findings, 1.0% l‐cysteine was selected for subsequent experiments due to its superior color preservation performance.

**TABLE 1:** Effect of different l‐cysteine concentrations on total color difference (Δ*E*) values of fresh‐cut quince slices during preliminary trials.

l‐cysteine solutions	ΔE
0.05%	23.89 ± 2.80
0.1%	20.91 ± 1.45
0.5%	18.71 ± 1.42
0.8%	12.34 ± 1.93
1.0%	11.18 ± 1.57

For the main experiments, quince slices were immersed for 1 min in 1% l‐cysteine or sodium metabisulfite solutions, while untreated samples served as controls. The pH values of the treatment solutions were measured as 5.20 for the l‐cysteine solution and 4.71 for the sodium metabisulfite solution. After draining for 30 min, samples were individually packaged in 50‐μm polyethylene bags and stored at 4°C for 9 days. Analyses were performed at 3‐day intervals, and all treatments were conducted in triplicate.

### 2.2 Color Determination

The surface color change of fresh‐cut quinces was quantified using a Colorimeter PCE‐CSM 7 (PCE Instruments, UK) to measure CIELAB color values (L*, a*, and b*). Color measurements were performed using a colorimeter calibrated with white and black standard plates prior to analysis. Measurements were taken from three different points of each sample, and the mean values were reported. In addition, color difference (Δ*E*) and browning index (BI) values were calculated from the L*, a*, and b* color parameters using the Equations (1), (2), (3).

(1) $$\mathrm{\Delta E}=\sqrt{{\left(L^{*}-L_{0}^{*}\right)}^{2}+{\left(a^{*}-a_{0}^{*}\right)}^{2}+{\left(b^{*}-b_{0}^{*}\right)}^{2}}$$

In the Equation (1), the subscript 0 represents the color values of the samples at Day 0, while the other values correspond to the color parameters measured during the storage period.

(2) $$X=\frac{a^{*}+1.75L^{*}}{5.645L^{*}+a^{*}-3.012b^{*}}$$

(3) $$\mathrm{BI}=\frac{100\left(X-0.31\right)}{0.17}$$

### 2.3 Enzyme Extraction and PPO Activity Determination

PPO activity was determined using a modified method of Cerit et al. (2020). Quince tissue (2.5 g) was homogenized for 30 s in 5 mL of 0.05 M phosphate buffer (pH 6.3) containing 1% PVP and 0.25% Triton X‐100 using a tissue homogenizer (Wiggen Hauser D130, Germany). The homogenate was filtered, centrifuged at 13,130*g* for 30 min (Hettich Universal 320R, Germany), and the supernatant was used as the enzyme extract. The sample cuvette consisted of 2.8 mL of phosphate buffer, 0.1 mL of substrate solution (0.1 mol/L catechol in phosphate buffer), and 0.1 mL of enzyme extract. Phosphate buffer was used instead of the enzyme extract in the blank cuvette. PPO activity was measured at 420 nm for 5 min using a UV–Vis spectrophotometer (Shimadzu UV‐1240, USA). One unit of PPO activity corresponded to an absorbance increase of 0.001 min^−1^.

### 2.4 Preparation of Extracts for TPC and Antioxidant Activities

Quince extracts for TPC and antioxidant activity were prepared according to Cerit et al. (2020) with minor modifications. Quince samples (2 g) were homogenized in 3 mL of methanol–water (75:25, v/v) for 3 min, sonicated for 15 min (Bandelin Sonorex RK 100H, Germany), and centrifuged at 13,130*g* for 10 min at 4°C. The extraction was repeated once; supernatants were combined, and the final volume was adjusted to 10 mL.

### 2.5 Determination of Antioxidant Activities and TPC

The 2,2‐diphenyl‐1‐picrylhydrazyl (DPPH) radical scavenging activity was determined using a modified method of Brand‐Williams et al. (1995) and Cerit et al. (2025). Briefly, 200 μL of extract was mixed with 3 mL of 0.051 mmol/L DPPH solution, incubated for 30 min at room temperature, and absorbance was measured at 517 nm. Results were expressed as mg Trolox equivalent (TE)/100 g sample. The calibration curve was prepared using Trolox standard solutions in the range of 0–80 ppm (*R* ^2^ = 0.998). FRAP was measured according to Benzie and Strain (1996). Extract (100 μL) was mixed with distilled water and FRAP reagent, incubated at 37°C for 15 min, and absorbance was read at 593 nm. Antioxidant capacity was expressed as FeSO_4_ equivalents (mg/100 g) using a calibration curve prepared with FeSO_4_ standard solutions in the range of 0–500 ppm (*R* ^2^ = 0.995). TPC was determined by the Folin–Ciocalteu method (Singleton et al. 1999) and expressed as g GAE/100 g sample. The calibration curve was prepared using gallic acid standard solutions in the range of 0–1000 ppm (*R* ^2^ = 0.998). All analyses were performed in triplicate.

### 2.6 Determination of GSH Contents

GSH levels in quince samples were determined using a chromatographic approach originally proposed by Winters et al. (1995) and later modified by Demirkol et al. (2004). First, samples were homogenized in a serine borate buffer solution (100 mM Tris–HCl, 10 mM borate, 5 mM serine, and 1 mM diethylenetriamine pentacetic acid, pH 7.0) and they were centrifuged at 2000*g* for 15 min; supernatants were used for the GSH content. The supernatants (250 μL) were derivatised with *N*‐(1‐pyrenyl)maleimide (NPM, 1 mM in acetonitrile) solution (750 μL). After 5 min at room temperature, 2 N HCl (10 μL) was added. The derivatized samples were injected into a Reliasil ODS‐1 C18 column (5 μμ, 250 × 4.6 mm) (Orochem, Naperville, USA) following filtering through a 0.45 nylon filter. The HPLC system (Hitachi, Tokyo, Japan) equipped with an L‐2130 pump, L‐2300 oven, L‐2200 autosampler, and 5440 fluorescence detector (excitation wavelength of 330 nm and an emission wavelength of 376 nm) was used. GSH level was calculated using a calibration curve (*R* ^2^ = 0.995). Results were expressed as nmol GSH per g of fresh quince. All analyses were performed in triplicate.

### 2.7 Statistical Analysis

The results were statistically analyzed by ANOVA using the SPSS software (version 15 for Windows, SPSS Inc., Chicago, IL, USA). Duncan's post hoc test was used to determine the differences between the groups (*p* ≤ 0.05). Statistical comparisons were performed among the samples within each storage period, and different letters indicated significant differences between treatments at the same storage day.

## 3 Results and Discussion

### 3.1 Effect of Treatment on Color Parameters

Color is a key quality attribute in fresh‐cut fruit products, directly influencing consumer preference and marketability. In this study, the effects of 1% l‐cysteine and 1% sulfite treatments on the color parameters (L*, a*, b*) of fresh‐cut quince slices were evaluated over 9 days of cold storage (Table 2). The findings highlight the distinct effectiveness of l‐cysteine compared to sulfite and untreated controls in mitigating enzymatic browning and preserving color integrity.

**TABLE 2:** Effect of l‐cysteine and sulfite on L*, a*, and b* color values, BI and Δ*E* in quince slices stored at 4°C for 9 days (*n* = 3).

Day	Treatment	L*	a*	b*	BI	Δ*E*
0	l‐cysteine	82.19 ± 0.46	−0.20 ± 0.05	32.92 ± 0.59	49.38 ± 1.46	—
	Sulfite	82.19 ± 0.46	−0.20 ± 0.05	32.92 ± 0.59	49.38 ± 1.46	—
	Untreated	82.19 ± 0.46	−0.20 ± 0.05	32.92 ± 0.59	49.38 ± 1.46	—
3	l‐cysteine	79.94 ± 0.75^b^	2.59 ± 0.27^b^	33.11 ± 0.78^b^	54.34 ± 1.44^b^	3.88 ± 0.40^c^
	Sulfite	82.38 ± 0.99^a^	−1.48 ± 0.14^c^	24.37 ± 0.66^c^	32.76 ± 0.60^c^	8.31 ± 0.66^b^
	Untreated	70.55 ± 0.47^c^	7.95 ± 0.72^a^	37.34 ± 0.84^a^	80.84 ± 2.08^a^	15.17 ± 0.33^a^
6	l‐cysteine	76.67 ± 0.68^b^	4.72 ± 0.78^b^	30.73 ± 0.16^a^	54.50 ± 0.36^b^	7.80 ± 0.12^b^
	Sulfite	80.54 ± 1.82^a^	−1.53 ± 0.21^c^	25.53 ± 2.01^b^	38.41 ± 2.14^c^	6.61 ± 0.91^b^
	Untreated	70.73 ± 1.21^c^	8.49 ± 0.22^a^	32.81 ± 0.87^a^	69.50 ± 1.43^a^	14.56 ± 1.08^a^
9	l‐cysteine	77.07 ± 0.82^a^	8.14 ± 0.35^a^	30.07 ± 0.63^b^	56.23 ± 1.45^b^	10.23 ± 0.16^b^
	Sulfite	75.33 ± 1.06^b^	−2.18 ± 0.10^c^	23.00 ± 1.35^c^	33.23 ± 2.39^c^	11.14 ± 0.43^ab^
	Untreated	73.92 ± 1.60^b^	6.45 ± 0.87^b^	34.45 ± 1.19^a^	67.30 ± 2.24^a^	11.88 ± 0.13^a^

*Note:* Different letters within each storage period indicate significant differences among treatments (*p* < 0.05).

The L* value, which represents lightness, decreased across all treatments during storage, indicating progressive browning. However, the decline was significantly (*p <* 0.05) less in the l‐cysteine‐treated group (from 82.19 to 77.07), compared to the untreated control (from 82.19 to 73.92). This suggests that l‐cysteine was effective in maintaining brightness throughout storage. Sulfite‐treated samples also retained higher L* values than the control (ending at 75.33), though not as effectively as l‐cysteine. Changes in the a* parameter (red‐green axis) further support these findings. While all groups exhibited increasing a* values—indicating a shift toward red and thus browning—the l‐cysteine‐treated slices showed a more controlled increase (from −0.20 to 8.14), compared to the untreated control (from −0.20 to 6.45). Sulfite treatment, on the other hand, was associated with declining a* values, suggesting the development of a greenish hue less desirable in fruit products. l‐cysteine mitigates browning primarily by inhibiting PPO activity. Its thiol (–SH) group reacts with quinones formed during phenolic oxidation, producing colorless adducts and preventing the formation of melanin‐like pigments (Dudley and Hotchkiss 1989; Molnár‐Perl and Friedman 1990). This mechanism, combined with l‐cysteine's antioxidant capacity, contributes to the observed preservation of both L* and a* values. In addition, l‐cysteine may slightly lower local pH, thereby further suppressing PPO and POD activity, both of which are pH‐sensitive enzymes involved in browning reactions (Cabezas‐Serrano et al. 2013). The b* values, representing the yellow‐blue axis, changed less dramatically but were also stabilized more effectively by l‐cysteine. This stabilization likely results from its antioxidant role in protecting yellow chromophores like flavonoids from oxidative degradation, thereby reducing non‐enzymatic browning (Nunes et al. 2017; Oliveira et al. 2011; Molnár‐Perl and Friedman 1990).

In addition to the individual L*, a*, and b* parameters, total color difference (Δ*E*) and BI values were evaluated to provide a more comprehensive assessment of color deterioration and browning progression during storage. The Δ*E* results demonstrated that there were noticeable color changes in all samples throughout storage; however, untreated samples exhibited significantly higher Δ*E* values, particularly on Days 3 and 6 (15.17 and 14.56, respectively), indicating more severe discoloration and browning development. In contrast, both l‐cysteine‐ and sulfite‐treated samples showed lower ΔE values than the untreated control during storage, suggesting improved preservation of the original color characteristics of quince slices. Similarly, BI values confirmed that untreated samples experienced the highest browning intensity during storage. The BI value of untreated quince slices increased markedly from 49.38 at Day 0 to 80.84 at Day 3, followed by a slight decrease during the later storage period, although values remained higher than those of treated samples. Sulfite treatment resulted in the lowest BI values throughout storage (32.76–38.41), indicating strong inhibition of browning reactions. l‐cysteine treatment also reduced browning development compared to the untreated control, with BI values ranging between 49.38 and 56.23. These findings support the color parameter results and demonstrate that both sulfite and l‐cysteine treatments effectively suppressed browning reactions and delayed color deterioration in fresh‐cut quince slices during cold storage.

When compared to the sharper decline in L* values and the less favorable shifts in a* in sulfite‐treated slices, l‐cysteine provided more consistent color retention. The current results are in agreement with previous studies. Zhang et al. (2023) reported that 1% l‐cysteine treatment in apple slices effectively maintained lightness and suppressed increases in a* and b* values during storage. Although the application methods differed—Zhang et al. (2023) applied l‐cysteine prior to slicing, whereas in our study it was applied post‐cutting—the consistent outcome highlights l‐cysteine's robustness in reducing enzymatic browning across different produce. Similarly, Cabezas‐Serrano et al. (2013) observed effective browning inhibition in minimally processed artichokes treated with l‐cysteine. In both studies, treated samples retained higher L* values and exhibited smaller changes in a* and b*, indicating broader applicability of l‐cysteine across botanical matrices. Finally, in our study, samples treated with l‐cysteine showed the best lightness preservation and the effective browning inhibition after 9 days of storage, which is consistent with the literature.

### 3.2 Effect of Treatment on PPO Activity

The impact of 1% l‐cysteine and 1% sodium metabisulfite treatments on PPO activity in quince slices during refrigerated storage was evaluated over a period of 9 days. PPO activity was measured on Days 0, 3, 6, and 9, and the results revealed significant differences based on both treatment type and storage duration (*p* < 0.05).

**FIGURE 1:**
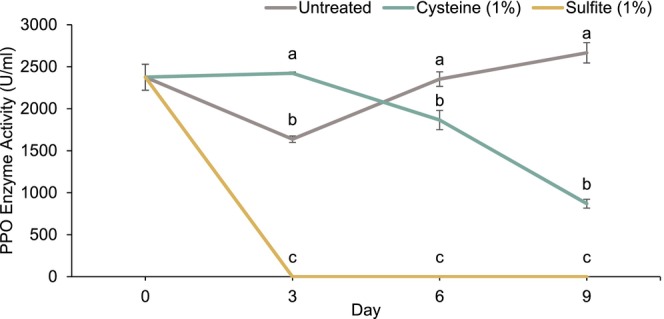
Effect of l‐cysteine and sulfite on PPO enzyme activities in quince slices stored at 4°C for 9 days (*n* = 3). Different letters within each storage period indicate significant differences among treatments (*p* < 0.05).

According to the ANOVA results, there was a strong interaction between treatment and storage time, indicating that PPO activity was influenced by how long the quinces were stored and which treatment was applied. The sodium metabisulfite‐treated samples exhibited the most consistent and effective inhibition of PPO activity throughout the storage period. PPO activity remained low and statistically unchanged between Days 3, 6, and 9, suggesting high efficacy and stability of sulfite as an anti‐browning agent (Figure 1).

In contrast, l‐cysteine demonstrated moderate inhibitory effects at the beginning of storage but gradually lost its effectiveness by Day 9. Although it reduced PPO activity compared to untreated samples, the enzyme activity increased significantly (*p <* 0.05) over time, indicating a decline in its protective capability.

Untreated samples showed a sharp increase in PPO activity over the storage period, with significantly higher values by Day 9. This trend aligns with previous studies that reported enhanced enzymatic browning due to wound‐induced activation of phenol metabolism in minimally processed fruits (Hu et al. 2022).

These results suggest that sulfite acts as a fast‐acting and sustained PPO inhibitor, whereas l‐cysteine's inhibitory effects are delayed and less complete. The rapid inhibition observed in the sulfite‐treated samples likely arises from its ability to react directly with quinones and prevent pigment formation, as well as irreversibly inactivating PPO's copper center, as described by Molnár‐Perl and Friedman (1990), Richard‐Forget et al. (1992) and Ali et al. (2018).

l‐cysteine's mode of action is believed to be reversible and redox‐dependent. It operates by reducing quinones back to diphenols and possibly forming colorless adducts with reactive intermediates. This mechanism, while beneficial, is less stable over time and more susceptible to oxidative degradation, which may explain the gradually increasing inhibition profile (Paulsen and Carroll 2013; Mailloux et al. 2014).

In comparing the inhibitory effects of l‐cysteine and sulfites on PPO activity, recent studies present compelling findings. Lim et al. (2019) demonstrated that l‐cysteine was significantly more effective in inhibiting PPO compared to other tested anti‐browning agents, achieving inhibition rates higher than those of sodium chloride and ascorbic acid. They reported a significant inhibition percentage using l‐cysteine, highlighting its potential as an effective inhibitor of enzymatic browning. Conversely, Sikora et al. (2019) found that sodium metabisulfite (20 mM) also significantly inhibited color development in PPO‐catalyzed reactions across various substrates. Their results indicated substantial reductions in enzymatic browning due to sodium metabisulfite. Additionally, Eidhin et al. (2006) reported that sodium metabisulfite was one of the most effective inhibitors when compared with other agents.

Collectively, while both l‐cysteine and sulfite compounds proved to be potent inhibitors of PPO, sodium metabisulfite exhibited superior inhibition in certain contexts, suggesting that their utilization could be complementary depending on specific application needs in food preservation. While sulfite is clearly more effective in inhibiting PPO, its use is limited by regulatory restrictions and allergenicity concerns. l‐cysteine, on the other hand, presents a food‐grade, safer alternative, though its moderate efficacy suggests that combinatory strategies or higher concentrations may be necessary to achieve inhibition levels comparable to sulfite.

The PPO activity data underscores the importance of choosing inhibitors based on the speed, strength, and duration of action required. For commercial quince products where browning control is critical during extended storage, sulfite remains the more reliable option. However, l‐cysteine's gradual inhibitory profile may be sufficient for short‐term freshness preservation in minimally processed applications.

### 3.3 Effect Treatment on the Antioxidant Activity

**FIGURE 2:**
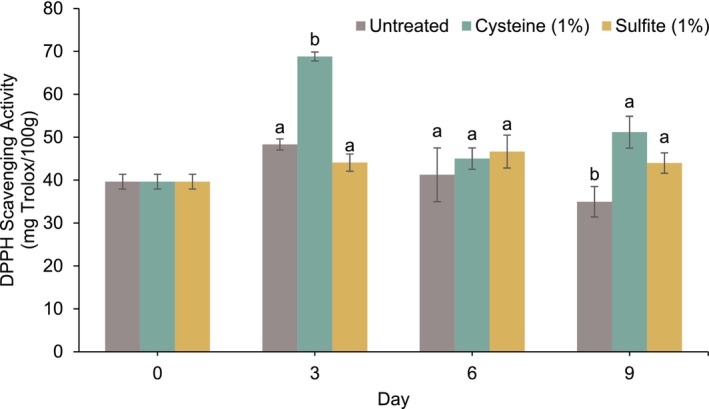
Effect of l‐cysteine and sulfite on DPPH scavenging activity in quince slices stored at 4°C for 9 days. Vertical bars represent ±SE of means (*n* = 3). Different letters within each storage period indicate significant differences among treatments (*p* < 0.05).

**FIGURE 3:**
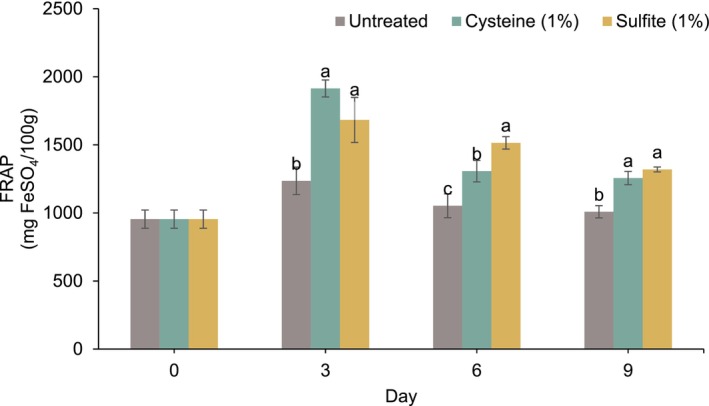
Effect of l‐cysteine and sulfite on FRAP in quince slices stored at 4°C for 9 days. Vertical bars represent ±SE of means (*n* = 3). Different letters within each storage period indicate significant differences among treatments (*p* < 0.05).

In this study, the antioxidant capacity of fresh‐cut quince slices was assessed using DPPH and FRAP assays. These two complementary methods evaluate distinct mechanisms of antioxidant action: DPPH measures the hydrogen‐donating ability of antioxidants, while FRAP assesses their electron‐donating capacity. Figures 2 and 3 show the DPPH and FRAP assay results. The combined use of these assays provides a comprehensive understanding of the redox behavior of quince under different treatments.

On Day 3 of storage, quince slices treated with 1% l‐cysteine exhibited a significantly (*p <* 0.05) higher DPPH radical scavenging activity (68.79 mg Trolox/100 g) compared to sulfite‐treated (48.32 mg Trolox/100 g) and untreated (44.07 mg Trolox/100 g) samples. This pronounced increase highlights l‐cysteine's effectiveness in enhancing antioxidant defenses in the early stages of storage. The sustained scavenging activity observed in l‐cysteine‐treated slices over the 9‐day period suggests robust antioxidant properties likely linked to its thiol‐based redox capacity and potential role in promoting endogenous antioxidant synthesis, such as GSH (Paulsen and Carroll 2013; Mailloux et al. 2014; Cerit et al. 2020). In contrast, stable DPPH activity was observed in sulfite‐treated slices.

Initial FRAP values (Day 0) were comparable across all treatments (940 mg FeSO_4_/100 g), indicating a similar baseline antioxidant potential. However, distinct differences emerged by day 3. l‐cysteine‐treated samples showed the highest FRAP activity (1940 mg FeSO_4_/100 g), followed by sulfite (1680 mg FeSO_4_/100 g), and untreated (1220 mg FeSO_4_/100 g) samples. These results demonstrate that l‐cysteine significantly enhances the fruit's reducing power, likely through increased ROS neutralization and stabilization of redox‐active compounds (Zhang et al. 2018; Mailloux et al. 2014).

Although FRAP activity in l‐cysteine‐treated slices declined slightly by Day 9 (1290 mg FeSO_4_/100 g), levels remained higher than both sulfite and control treatments, suggesting sustained antioxidant protection despite ongoing oxidative stress. Sulfite‐treated samples experienced a similar decline (to 1340 mg FeSO_4_/100 g), consistent with known limitations in sulfite stability over time (Vally et al. 2009; D'Amore et al. 2020).

The untreated group exhibited the lowest FRAP values across all time points, decreasing from 1220 mg FeSO_4_/100 g at Day 3 to 1020 mg FeSO_4_/100 g at Day 9, reflecting the progression of oxidative degradation in the absence of antioxidant intervention (Moon et al. 2020; Meitha et al. 2020).

The enhanced and sustained antioxidant activity observed with l‐cysteine treatment across both DPPH and FRAP assays underscores its potential as an effective natural preservative. l‐cysteine's thiol functionality supports both non‐enzymatic and enzymatic antioxidant pathways, offering superior redox flexibility compared to sulfite. Prior studies in fruits such as litchi and plums have similarly reported improved antioxidant enzyme activity and reduced oxidative damage following l‐cysteine application (Ali et al. 2016; Banin Sogvar et al. 2020).

Interestingly, the FRAP assay demonstrated greater sensitivity in detecting early antioxidant changes than DPPH, particularly, in cysteine‐treated samples. This observation is consistent with previous findings in plum fruit, where FRAP was more responsive to l‐cysteine treatments (Banin Sogvar et al. 2020). These results affirm FRAP's suitability for evaluating cumulative oxidative impacts during storage. In conclusion, based on the findings of both analyses in our study, l‐cysteine application was found to be at least as effective as sulfite application in preserving antioxidant activity under both short‐term (3 days) and long‐term (9 days).

### 3.4 Effect of Treatment on TPC

**FIGURE 4:**
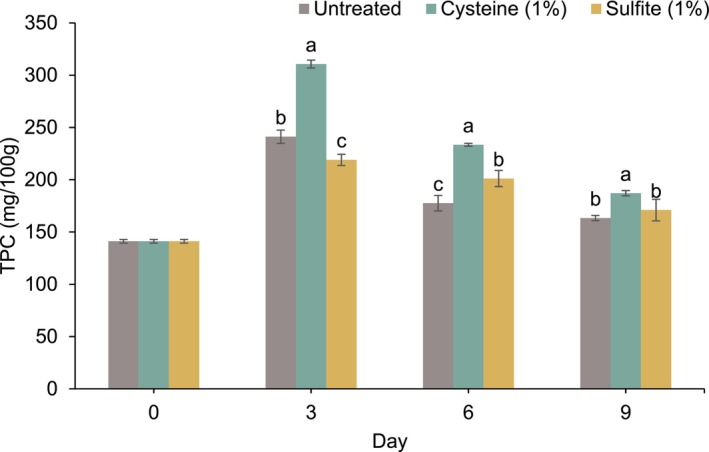
Effect of l‐cysteine and sulfite on TPC in quince slices stored at 4°C for 9 days. Vertical bars represent ±SE of means (*n* = 3). Different letters within each storage period indicate significant differences among treatments (*p* < 0.05).

The TPC, a critical indicator of antioxidant potential and oxidative stability in fresh‐cut fruit, showed significant variation during the 9‐day storage period in quince slices subjected to different treatments (1% l‐cysteine, 1% sodium metabisulfite and control). Figure 4 shows the TPC assay results.

On Day 3, quince slices treated with l‐cysteine exhibited the highest TPC content (310 mg/100 g), enhancing phenolic levels relative to untreated (240 mg/100 g) and sulfite‐treated slices (220 mg/100 g). The noted increase (*p* < 0.05) in TPC associated with l‐cysteine aligns with its role as a reactive thiol that can prevent the oxidation of phenolic compounds, thereby preserving the phenolic profile (Paulsen and Carroll 2013; Zhang et al. 2018; Moon et al. 2020).

On Days 6 and 9, the TPC values of l‐cysteine‐treated slices remained relatively high (230 and 180 mg/100 g, respectively), despite a slight decline. This gradual decrease may indicate ongoing oxidative processes, but the treated samples demonstrated a significant advantage in phenolic content compared to untreated slices, which showed a steeper decline from 240 mg/100 g on Day 3 to 165 mg/100 g on Day 9 (Meitha et al. 2020).

Sulfite treatment also positively impacted the TPC, with values registered at 220 mg/100 g on Day 3, remaining relatively stable at 200 mg/100 g on Day 6 and dropping slightly to 170 mg/100 g by Day 9. This reduced efficacy might stem from sulfite volatility or its reactivity being limited to early storage stages.

In contrast, the untreated control samples exhibited the lowest TPC levels, with a significant (*p* < 0.05) reduction from 240 mg/100 g on Day 3 to 165 mg/100 g by Day 9. This reduction highlights the detrimental effect of enzymatic browning processes on phenolic content when no preservative measures are applied, confirming that reactive intermediates lead to phenol oxidation and loss (Zhu et al. 2022).

In the current study, l‐cysteine's superior performance over sulfite is particularly notable given its sulfur‐based structure, which enables it to act similarly to sulfites in oxidative stress mitigation but with potentially lower health risks (Moon et al. 2020; Banin Sogvar et al. 2020; Pace et al. 2014). Moreover, l‐cysteine has been found to upregulate antioxidant enzymes and stabilize cellular membranes, thereby further delaying degradation of phenolic compounds (Jiang et al. 2022).

Importantly, the use of sulfites in food has raised safety concerns, including allergenic reactions in sensitive individuals, leading to their regulation or restriction in many countries. Therefore, identifying safer yet equally effective alternatives such as l‐cysteine is of great interest (Ali et al. 2016; Banin Sogvar et al. 2020).

Several recent studies have highlighted the advantages of l‐cysteine over both sulfite treatments and untreated controls in maintaining phenolic compounds during postharvest storage. For example, Gohari et al. (2021) demonstrated that applying 0.4% l‐cysteine effectively preserved TPC and significantly reduced internal browning in peaches stored at low temperatures. Similarly, research by Ali et al. (2016) revealed that l‐cysteine treatment suppressed both PPO and POD enzymatic activities in litchi fruit, resulting in higher retention of total phenolics and delayed browning of the pericarp. Banin Sogvar et al. (2020) also reported that 0.5% l‐cysteine enhanced antioxidant potential and total phenolic preservation in plums under cold storage conditions. In conclusion, our study confirmed the literature findings that the best preservation of TPC throughout the entire storage period was achieved with the application of l‐cysteine.

### 3.5 Effect of Treatment on GSH Content

**FIGURE 5:**
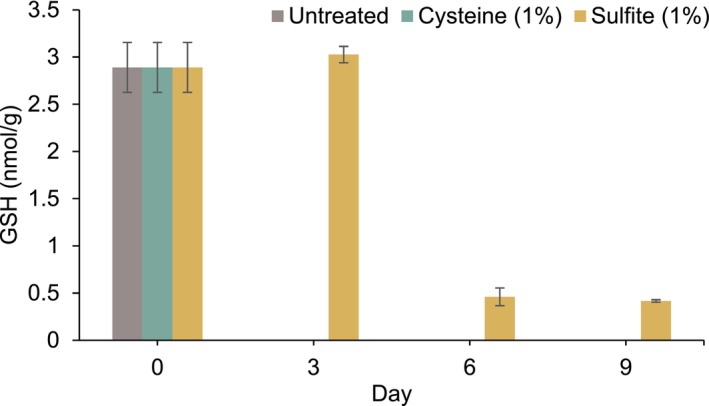
Effect of l‐cysteine and sulfite on GSH in quince slices stored at 4°C for 9 days. Vertical bars represent ± SE. of means (*n* = 3).

The amount of GSH, a tripeptide vital for maintaining oxidative balance in treated and untreated quince slices, is shown in Figure 5. At Day 0, all treatment groups exhibited similar GSH levels (2.9 nmol/g). However, as storage progressed, significant (*p* < 0.05) differences emerged. By Day 3, GSH was undetectable in both the untreated and l‐cysteine‐treated samples, whereas sulfite‐treated slices retained a measurable GSH level of 3.05 nmol/g. This suggests that sulfite treatment supports antioxidant capacity, likely by preserving reduced thiol groups and mitigating oxidative damage, as reported in previous studies (D'Amore et al. 2020). Studies investigating the impact of sulfites on GSH levels in fruits and vegetables are limited; the majority of available data are derived from enological studies. In winemaking, the relationship between sulfite addition and GSH levels is complex, potentially yielding both beneficial and detrimental effects depending on the context (Fracassetti et al. 2016; Arapitsas et al. 2016; Du Toit et al. 2007).

Despite this initial increase, GSH levels in sulfite‐treated samples gradually declined to 0.5 nmol/g on Day 6 and 0.4 nmol/g on Day 9, indicating progressive depletion under extended oxidative stress. Prolonged ROS exposure is known to exhaust GSH pools due to its intensive use in scavenging activities (Meitha et al. 2020).

In contrast, the sustained absence of GSH in untreated and l‐cysteine‐treated samples throughout the storage period suggests rapid depletion or oxidation of GSH, likely due to enhanced enzymatic browning and PPO activity (Moon et al. 2020; Ali et al. 2014). While l‐cysteine is a precursor for GSH synthesis and can stabilize thiol groups, its 1% application did not preserve GSH in quince slices, possibly due to rapid GSH turnover or l‐cysteine's pro‐oxidant behavior in this fruit matrix. It can enhance antioxidant defenses but may also generate ROS through redox cycling and Fenton‐type reactions, particularly in the presence of transition metals like Fe^2+^ and Cu^2+^ (Forman et al. 1983; Haag et al. 2012; Szalai et al. 2009; Ali et al. 2016). In our study, the mechanical damage from slicing quince may contribute to the inadequate levels of l‐cysteine needed for effective GSH protection. Literature indicates that a low‐concentration cysteine coating (e.g., 0.05%) is sufficient for the storage of goji berries, which are small, intact fruits (Wang et al. 2022). This finding highlights the necessity of considering tissue integrity and processing concentration when developing postharvest antioxidant strategies.

To summarize, while a 1% concentration of metabisulfite was sufficient to preserve GSH during short‐term storage of sliced quince, the same effect was not observed with l‐cysteine at this concentration. This is vital for preserving fruit quality, as GSH not only neutralizes ROS but also contributes to the regeneration of other antioxidants via the ascorbate–GSH cycle. Nevertheless, the continued use of sulfites in food systems is under regulatory scrutiny due to potential health concerns. Therefore, developing alternative strategies—such as encapsulated or stabilized l‐cysteine systems—may offer safer approaches to mimic the thiol‐preserving effects of sulfites without their associated toxicological risks.

### 3.6 Correlations Among BI, PPO, TPC, FRAP, DPPH, and GSH

Table 3 presents the Pearson correlation coefficients among BI, PPO, TPC, FRAP, DPPH, and GSH parameters. A strong positive and statistically significant correlation was observed between BI and PPO (*r* = 0.690, *p* < 0.001). In contrast, BI showed significant negative correlations with FRAP (*r* = −0.425, *p* < 0.05) and GSH (*r* = −0.484, p < 0.001). No significant correlations were detected between BI and TPC or DPPH.

**TABLE 3:** Pearson's correlation coefficients among BI, PPO, TPC, FRAP, DPPH, and GSH.

	BI	PPO	TPC	FRAP	DPPH	GSH
BI	—	0.690***	0.112	−0.425*	−0.117	−0.484**
PPO	—	—	0.064	−0.372*	−0.142	0.141
TPC	—	—	—	0.780***	0.760***	−0.352
FRAP	—	—	—	—	0.810***	−0.104
DPPH	—	—	—	—	—	−0.067
GSH	—	—	—	—	—	—

* *p* < 0.05.

** *p* < 0.01.

*** *p* < 0.001.

PPO exhibited a moderate negative correlation with FRAP (*r* = −0.372, *p <* 0.05), whereas its correlations with TPC, DPPH, and GSH were not statistically significant. Strong positive correlations were identified between TPC and FRAP (*r* = 0.780, *p <* 0.001), as well as between TPC and DPPH (*r* = 0.760, *p* < 0.001). Similarly, FRAP and DPPH were strongly and positively correlated (*r* = 0.810, *p <* 0.001). These findings indicate that increased TPC is associated with enhanced antioxidant capacity. Similarly, in one of the studies, it was reported a strong correlation between TPC and FRAP values in pepper samples (*R* ^2^ = 0.7494). However, unlike those findings, our results also demonstrated a strong positive correlation between TPC and DPPH values, suggesting that phenolic compounds played an important role in free radical scavenging activity in the analyzed samples (Cerit et al. 2025). Among the evaluated parameters, GSH did not show significant correlations with most variables, except for a significant negative correlation with BI. This may be attributed to the fact that GSH levels were below the detection limit in most samples, which may have limited the ability of the correlation analysis to fully reflect the actual relationships among variables.

## 4 Conclusion

This study demonstrated that l‐cysteine is an effective and promising natural alternative to traditional sulfite‐based preservatives for reducing enzymatic browning and preserving antioxidant qualities in fresh‐cut quince. Compared to sodium metabisulfite, l‐cysteine treatment better maintained visual quality by retaining lightness (L*), limiting color shifts (a*, b*) and enhancing antioxidant activity, particularly in the early to mid‐storage period. Additionally, l‐cysteine significantly improved TPC, suggesting a protective effect against oxidative degradation.

However, while l‐cysteine showed strong initial effects, its capacity to maintain GSH levels diminished over time, unlike sulfite, which preserved GSH more effectively throughout storage. This indicates that although l‐cysteine is safer and more consumer‐friendly, its protective mechanisms may be less stable under prolonged oxidative stress.

In conclusion, l‐cysteine offers a viable, food‐grade strategy for inhibiting browning and maintaining the nutritional and sensory quality of fresh‐cut quince. Its application could contribute to cleaner‐label processing and meet increasing consumer demand for safer food preservatives. Future work may explore optimizing concentration, combining with other natural compounds, or using encapsulated forms to enhance long‐term efficacy.

## Author Contributions

**İnci Cerit:** investigation, writing – original draft, formal analysis. **Özlem Aktürk Gümüşay:** formal analysis, writing – original draft. **Omca Demirkol:** conceptualization, methodology, writing – original draft, writing – review and editing, resources, supervision, investigation.

## Funding

The authors have nothing to report.

## Ethics Statement

The authors have nothing to report.

## Conflicts of Interest

The authors declare no conflicts of interest.

## Data Availability

The data that support the findings of this study are available from the corresponding author upon reasonable request.
